# Vickybot, a Chatbot for Anxiety-Depressive Symptoms and Work-Related Burnout in Primary Care and Health Care Professionals: Development, Feasibility, and Potential Effectiveness Studies

**DOI:** 10.2196/43293

**Published:** 2023-04-03

**Authors:** Gerard Anmella, Miriam Sanabra, Mireia Primé-Tous, Xavier Segú, Myriam Cavero, Ivette Morilla, Iria Grande, Victoria Ruiz, Ariadna Mas, Inés Martín-Villalba, Alejandro Caballo, Julia-Parisad Esteva, Arturo Rodríguez-Rey, Flavia Piazza, Francisco José Valdesoiro, Claudia Rodriguez-Torrella, Marta Espinosa, Giulia Virgili, Carlota Sorroche, Alicia Ruiz, Aleix Solanes, Joaquim Radua, María Antonieta Also, Elisenda Sant, Sandra Murgui, Mireia Sans-Corrales, Allan H Young, Victor Vicens, Jordi Blanch, Elsa Caballeria, Hugo López-Pelayo, Clara López, Victoria Olivé, Laura Pujol, Sebastiana Quesada, Brisa Solé, Carla Torrent, Anabel Martínez-Aran, Joana Guarch, Ricard Navinés, Andrea Murru, Giovanna Fico, Michele de Prisco, Vicenzo Oliva, Silvia Amoretti, Casimiro Pio-Carrino, María Fernández-Canseco, Marta Villegas, Eduard Vieta, Diego Hidalgo-Mazzei

**Affiliations:** 1 Department of Psychiatry and Psychology Institute of Neuroscience Hospital Clínic de Barcelona Barcelona Spain; 2 Bipolar and Depressive Disorders Unit Digital Innovation Group Institut d'Investigacions Biomèdiques August Pi i Sunyer (IDIBAPS) Barcelona Spain; 3 Biomedical Research Networking Centre Consortium on Mental Health (CIBERSAM) Instituto de Salud Carlos III Madrid Spain; 4 Department of Medicine School of Medicine and Health Sciences University of Barcelona (UB) Barcelona Spain; 5 Institute of Neurosciences (UBNeuro) Barcelona Spain; 6 Imaging of Mood- and Anxiety-Related Disorders group (IMARD) Institut d'Investigacions Biomèdiques August Pi i Sunyer (IDIBAPS) Barcelona Spain; 7 Early Psychosis: Interventions & Clinical-detection (EPIC) Lab, Department of Psychosis Studies Institute of Psychiatry Psychology and Neuroscience King's College London London United Kingdom; 8 Center for Psychiatry Research Department of Clinical Neuroscience Karolinska Institutet Stockholm Sweden; 9 CAP Casanova, Consorci d'Atenció Primaria de Salut Barcelona Esquerra (CAPSBE) Barcelona Spain; 10 CAP Borrell, Consorci d'Atenció Primaria de Salut Barcelona Esquerra (CAPSBE) Barcelona Spain; 11 Centre for Affective Disorders Institute of Psychiatry, Psychology & Neuroscience King's College London London United Kingdom; 12 Abi Global Health Dublin Ireland; 13 European Association of Psychosomatic Medicine Oregon, OR United States; 14 Grup de Recerca en Addicions Clínic Department of Psychiatry Addiction Unit Hospital Clínic of Barcelona Barcelona Spain; 15 Red de Investigación en Atención Primaria de Adicciones (RIAPAD) Barcelona Spain; 16 Occupational Health Department, Hospital Clínic de Barcelona Barcelona Spain; 17 Barcelona Supercomputing Center (BSC) Text Mining Technologies in the Health Domain Barcelona Spain

**Keywords:** primary care, health care workers, depression, anxiety, symptom, burnout, digital, smartphone, chatbot, primary care digital support tool in mental health, PRESTO

## Abstract

**Background:**

Many people attending primary care (PC) have anxiety-depressive symptoms and work-related burnout compounded by a lack of resources to meet their needs. The COVID-19 pandemic has exacerbated this problem, and digital tools have been proposed as a solution.

**Objective:**

We aimed to present the development, feasibility, and potential effectiveness of Vickybot, a chatbot aimed at screening, monitoring, and reducing anxiety-depressive symptoms and work-related burnout, and detecting suicide risk in patients from PC and health care workers.

**Methods:**

Healthy controls (HCs) tested Vickybot for reliability. For the simulation study, HCs used Vickybot for 2 weeks to simulate different clinical situations. For feasibility and effectiveness study, people consulting PC or health care workers with mental health problems used Vickybot for 1 month. Self-assessments for anxiety (Generalized Anxiety Disorder 7-item) and depression (Patient Health Questionnaire-9) symptoms and work-related burnout (based on the Maslach Burnout Inventory) were administered at baseline and every 2 weeks. Feasibility was determined from both subjective and objective user-engagement indicators (UEIs). Potential effectiveness was measured using paired 2-tailed *t* tests or Wilcoxon signed-rank test for changes in self-assessment scores.

**Results:**

Overall, 40 HCs tested Vickybot simultaneously, and the data were reliably transmitted and registered. For simulation, 17 HCs (n=13, 76% female; mean age 36.5, SD 9.7 years) received 98.8% of the expected modules. Suicidal alerts were received correctly. For the feasibility and potential effectiveness study, 34 patients (15 from PC and 19 health care workers; 76% [26/34] female; mean age 35.3, SD 10.1 years) completed the first self-assessments, with 100% (34/34) presenting anxiety symptoms, 94% (32/34) depressive symptoms, and 65% (22/34) work-related burnout. In addition, 27% (9/34) of patients completed the second self-assessment after 2 weeks of use. No significant differences were found between the first and second self-assessments for anxiety (*t*_8_=1.000; *P*=.34) or depressive (*t*_8_=0.40; *P*=.70) symptoms. However, work-related burnout scores were moderately reduced (*z*=−2.07, *P*=.04, *r*=0.32). There was a nonsignificant trend toward a greater reduction in anxiety-depressive symptoms and work-related burnout with greater use of the chatbot. Furthermore, 9% (3/34) of patients activated the suicide alert, and the research team promptly intervened with successful outcomes. Vickybot showed high subjective UEI (acceptability, usability, and satisfaction), but low objective UEI (completion, adherence, compliance, and engagement). Vickybot was moderately feasible.

**Conclusions:**

The chatbot was useful in screening for the presence and severity of anxiety and depressive symptoms, and for detecting suicidal risk. Potential effectiveness was shown to reduce work-related burnout but not anxiety or depressive symptoms. Subjective perceptions of use contrasted with low objective-use metrics. Our results are promising but suggest the need to adapt and enhance the smartphone-based solution to improve engagement. A consensus on how to report UEIs and validate digital solutions, particularly for chatbots, is required.

## Introduction

Between 30% and 50% of people attending primary care (PC) in Spain have mental health problems, with a large majority demonstrating mild-to-moderate anxiety and depressive symptoms, usually work-related [1,2]. Only 5% of these people will eventually require specialized mental health care [3]. Nevertheless, access to specialized mental health care can take up to 3 months because of the high number of referrals and lack of resources [4-6], including personnel, consultation times, and lack of training of general practitioners (GPs) to address mental health problems [7]. Considering this complex and problematic challenge, during the last decade, the Spanish National Health System has launched a Primary Care Mental Health Support Program (PCMHSP) composed of mental health specialists, including psychiatrists, psychologists, and nurses, who dedicate part of their workweek to PC [8]. Nonetheless, the care demand still far exceeds the resources currently available at PC, representing a global challenge across the international community [9,10]. Consequently, collaborative initiatives are increasingly proposed to address this issue [11].

The unresolved delay in receiving specialized attention added to the lack of resources in PC, may lead to an increased and unnecessary risk of exacerbation of symptoms, affecting the quality of life of patients and potentially increasing sick leaves, and, in the worst-case scenario, irreparable consequences such as suicide [12]. Evidence shows that up to 30% of patients with depression reported not receiving any type of care in the previous year [13]. This situation has led to a substantial increase in the unnecessary and premature prescription of psychotropic drugs (up to 50% of people attending PC) [14-17], and superfluous referrals to specialized care and a lack of early detection of severe mental health problems.

In addition to this already challenging situation, the COVID-19 pandemic has caused a considerable impact on the mental health of the general population, with increases of >25% in both anxiety and depressive disorders [18]. This has exacerbated the existing problems of mental health care in PC [19-21], and also impacted health care workers, especially those in the front line [22-25]. In particular, health care workers have reported increased rates of anxiety, depression, and posttraumatic stress disorder (PTSD) symptoms [26-28], with potentially severe mental health consequences [25,29,30]. This increase in mental health problems in health care workers is related to an overload of work and includes symptoms such as mental and physical exhaustion, depersonalization, diminished sense of personal accomplishment, and reduced professional functioning, thus fulfilling the criteria of work-related burnout [31,32], which is usually comorbid with symptoms of anxiety and depression such as fatigue, insomnia, and sadness [33,34]. In fact, work-related burnout is a major issue among the general population, particularly in health care workers [35], which can lead to increased sick leaves with a severe economic impact [2], or even major consequences, such as suicide.

This sudden worldwide increase in the demand for mental health care has proven challenging, with essential mental health services being disrupted [36]. Digital psychological tools have been proposed as a potential solution [37-39]. In fact, some digital interventions through smartphone apps have proven effective in improving anxiety [40], depressive symptoms [41], and work-related burnout [42-44], especially in PC settings [45,46], and also for people with severe mental disorders such as bipolar disorder [47,48].

More recently, chatbots (virtual assistants with artificial intelligence), by directly implementing Natural Language Processing (NLP) techniques and powered by artificial intelligence, have been shown to increase engagement, usability, and effectiveness of these interventions by offering a more friendly and personalized approach to users [49,50]. In people with PTSD (eg, war veterans), the anonymous nature of chatbots has also effectively increased the disclosure of traumatic events [51]. This may be of particular interest to health care workers who have been exposed to extreme and continuous pressure during the COVID-19 pandemic. In addition, NLP techniques have proven useful for both the design of smartphone apps [52] and for identifying differential patient profiles to tailor specific psychological interventions according to these characteristics [53]. Apps and chatbots allow continuous psychological intervention that adapts to the needs, schedules, and status of the patient in a dynamic way based on the progression of the patient, with the possibility of capturing active or passive data related to symptom evolution [54,55].

The primary care digital support tool in mental health (PRESTO) project [56] intends to mitigate the growing problem of mental health problems in PC and among health care workers by developing a digital decision support platform combining machine-learning severity prediction models (phase 1) with a smartphone-based app for screening, monitoring, and delivering evidence-based psychological interventions to reduce anxiety, depressive symptoms, and work-related burnout (phase 2).

In this work, we present the pilot results of phase 2: the development and studies of feasibility and potential effectiveness in PC patients and health care workers. The primary aim of this study was to assess the feasibility of the intervention. Secondary aims included assessing (1) its use in screening and monitoring anxiety and depressive symptoms and work-related burnout, (2) the potential effectiveness of the intervention in reducing them, and (3) the ability of the intervention to detect suicide risk.

## Methods

### The “Vickybot” Intervention: Development and Functions

**Figure 1:**
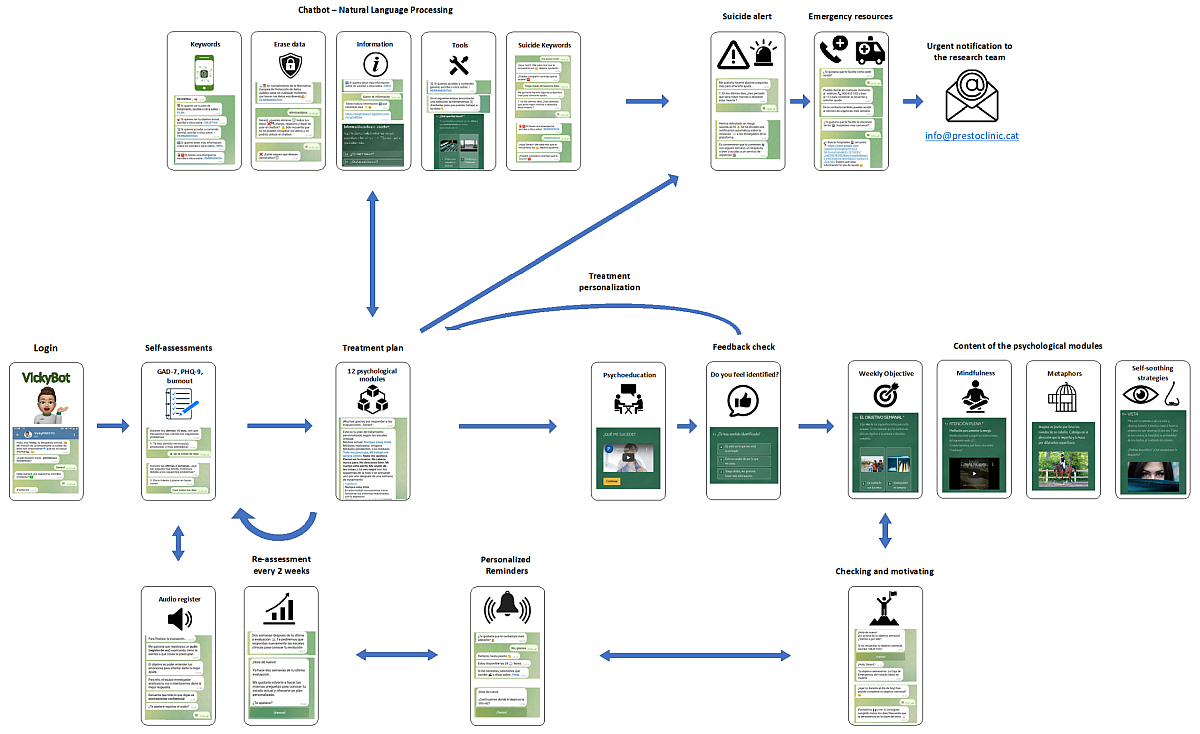
Scheme of the main functions of the intervention Vickybot. Please see Multimedia Appendix 1 for a larger version.

We developed a chatbot intended to screen and monitor anxiety and depressive symptoms and work-related burnout while providing evidence-based psychological interventions. User-centered development strategies have been adopted with evidence of improved user retention and satisfaction with the end product and increased chances of engagement in long-term real-world clinical settings [57]. Considering the preferences and recommendations of patients and health care workers presented in the protocol [56], the research team outlined the priority aspects of the digital tool as well as the psychological contents to be included. Its main functions included were as follows (Figure 1):

1. Self-administered scales for screening and monitoring anxiety (General Anxiety Disorder-7 [GAD-7] [58,59]), depressive symptoms (Patient Health Questionnaire-9 [PHQ-9] [60,61]), and work-related burnout (evaluated through 2 questions based on the emotional exhaustion dimension items from the Maslach Burnout Inventory [62,63]); users were prompted to complete all self-assessments every 2 weeks.
2. Psychological modules according to self-assessment; different modules were recommended for each individual according to an algorithm that considered the severity of the scales’ scores (both global and item-based). There were 12 short, sequential, and customizable modules: 10 modules for anxiety and depressive symptoms (1: depressed mood, 2: anxiety, 3: apathy-anhedonia, 4: depressive cognitions, 5: suicidal thoughts, 6: restlessness, 7: decreased concentration, 8: overthinking, 9: irritability, and 10: sleep disturbance), and 2 modules for the management of the work-related stress (11) and burnout (12). The psychological modules are based on eclectic therapy [64], mostly including cognitive behavioral therapy (CBT) techniques [65,66], mindfulness, dialectical behavioral therapy [67], and acceptance and commitment therapy [68]. Specific functions included psychoeducation and weekly objectives (based on CBT), mindfulness (based on the stress-reduction program [69]), self-soothing strategies (based on dialectical behavioral therapy), and metaphors (based on acceptance and commitment therapy). All modules started with the psychoeducation section to evaluate whether participants identified with the symptoms and situations presented, and afterward the other functions were shown. Then, participants could navigate among the aforementioned functions and use those that they felt more comfortable with or found more useful. Modules 1 and 2 (depressed mood and anxiety) included only psychoeducation. Module 4 (depressive cognitions) included an intervention for people experiencing a negative view of either the self, the world, and the future. These cognitions include failure, rejection, loss, guilt, lack of self-confidence or self-esteem, hopelessness, helplessness, and a lack of hope for improvement.
3. A chatbot system allowing users to navigate through the intervention in a friendly manner and enabling access the former functions, according to the scenarios presented by each user, while also being able to answer questions and detect emergency situations, such as suicidal thoughts.
4. Emergency alert for suicidal thoughts; for users who scored item 9 of the PHQ-9 (suicidal thoughts) or if the chatbot detected suicidal inputs using NLP, an alert was sent to the research team and the user was recommended to immediately visit the emergency department and provided with emergency resources (telephone number for health emergencies and nearby hospital locations).
5. Reminder for the “weekly objective” function from the modules; users can choose an hour of the day to perform the directed activity, and they have the opportunity to create an automatic notification. Other reminders were set for self-assessment monitoring every 2 weeks and to assess user experience with the intervention after 1 month of use.
6. Audio register; after completing the self-assessments, the participants were offered to register an audio file about their feelings or concerns regarding future voice analysis.

### Feasibility and Potential Effectiveness Evaluation of the Intervention

To consolidate the development and evaluate the feasibility and potential effectiveness of the intervention, the following phases were conducted sequentially.

#### Setup Phase

Over the course of 2 weeks, 40 healthy controls (HCs) used Vickybot and were asked to test the different functionalities without specific instructions (eg, responding to self-assessments, accessing the psychological modules, setting reminders, etc). A technical test was performed to evaluate the stability and reliability of the data transmission between devices and servers. The tolerance of calls per minute to the server was verified, and logs of bugs were also collected. HCs were recruited via advertisements, and psychiatric disorders were excluded through semistructured interviews.

#### Simulation Phase

A total of 17 HCs, all participants from the previous setup phase, were instructed to use the Vickybot for 2 more weeks and simulate different clinical situations indicated by the research team to elicit specific functions from the chatbot. Clinical simulations were designed to identify specific clinical profiles (severe-moderate-mild anxiety and depressive symptoms, work-related burnout, isolated symptoms, suicidal thoughts, etc), as shown in Table 1. The data recorded on the server were correlated with the patterns identified in each clinical situation. In addition, the treatment algorithm for customizing the psychological modules was tested and calibrated for each simulation. Finally, the baseline patterns of each simulator were compared with their own follow-up outcomes as a function of time to assess variability and longitudinal registers. After the simulation phase, the HCs were asked for their insights into the functions that they thought could be improved. Subjective user-engagement indicators (UEI; acceptability, usability, and satisfaction) were assessed using the Mobile Health App Usability Questionnaire (MAUQ) [70,71] (Multimedia Appendix 2), which includes 18 statements to determine the usability, acceptability, and satisfaction of mental health interventions.

**Table 1:** Clinical simulations and functions of the intervention assessed.

User	Clinical situation to be simulated	Specific functions to be tested	Specific psychological modules to be activated
1	Severe anxiety symptoms	Self-assessments, treatment algorithm, modules	2. Anxiety; 8. Overthinking; 9. Irritability; and 10. Sleep disturbance
2	Moderate anxiety symptoms	Self-assessments, treatment algorithm, modules	2. Anxiety; 8. Overthinking; 9. Irritability; and 10. Sleep disturbance
3	Severe depressive symptoms	Self-assessments, treatment algorithm, modules	1. Depressed mood; 3. Apathy-anhedonia; 4. Depressive cognitions; 5. Suicidal thoughts; 6. Restlessness; 7. Decreased concentration; and 10. Sleep disturbance
4	Moderate depressive symptoms without suicidal thoughts	Self-assessments, treatment algorithm, modules	1. Depressed mood; 3. Apathy-anhedonia; 4. Depressive cognitions; 5. Suicidal thoughts; 6. Restlessness; 7. Decreased concentration; and 10. Sleep disturbance
5	Work-related burnout with moderate anxiety symptoms	Self-assessments, treatment algorithm, modules	2. Anxiety; 8. Overthinking; 9. Irritability; 10. Sleep disturbance; 11. Work-related stress; and 12. Burnout
6	Work-related burnout with moderate depressive symptoms	Self-assessments, treatment algorithm, modules	11. Work-related stress and 12. Burnout
7	Depressive symptoms with suicidal thoughts	Emergency alerts of suicidal thoughts (item-9 of the PHQ-9^a^ and chatbot NLP^b^-intent detection), automatic alert to the research team	5. Suicidal thoughts
8	Lack of anxiety-depressive symptoms and lack of work-related burnout	Tools and “null” function of the treatment algorithm	N/A^c^
9	Insomnia (solely)	Self-assessments, treatment algorithm, modules	10. Sleep disturbance
10	Recurring concerns (solely)	Self-assessments, treatment algorithm, modules	8. Overthinking
11	Cognitive problems (solely)	Self-assessments, treatment algorithm, modules	7. Decreased concentration
12	Irritability (solely)	Self-assessments, treatment algorithm, modules	9. Irritability
13	Depressive cognitions such as guilt or lack of confidence or self-esteem (solely)	Self-assessments, treatment algorithm, modules	4. Depressive cognitions
14	Apathy (solely)	Self-assessments, treatment algorithm, modules	3. Apathy-anhedonia
15	Insomnia and irritability (both solely)	Self-assessments, specific combination of the treatment algorithm, modules	9. Irritability and 10. Sleep disturbance
16	Depressive cognitions and recurring concerns (both solely)	Self-assessments, specific combination of the treatment algorithm, modules	4. Depressive cognitions and 8. Overthinking
17	User with erratic behavior (contradictory self-assessment feedback, insults, skips the proposed modules, etc)	Self-assessments, treatment algorithm, modules; insult intent	N/A

^a^PHQ-9: Patient Health Questionnaire-9.

^b^NLP: Natural Language Processing.

^c^N/A: not applicable.

#### Feasibility and Potential Effectiveness Study: Participants, Procedure, and Measures

Inclusion criteria were as follows: (1) people referred to the PCMHSP from the GP or health care workers from Hospital Clínic de Barcelona referred to specialized mental health care from the Department of Occupational Health, (2) aged between 18 and 65 years, (3) have a compatible smartphone and sufficient digital skills to use a chatbot (access, main functions use, etc), and (4) have accepted and signed the informed consent for study participation. Exclusion criteria were as follows: (1) previous or present diagnosis of a severe mental disorder and (2) risk of suicide detected during clinical consultations before the use of the chatbot.

**Figure 2:**
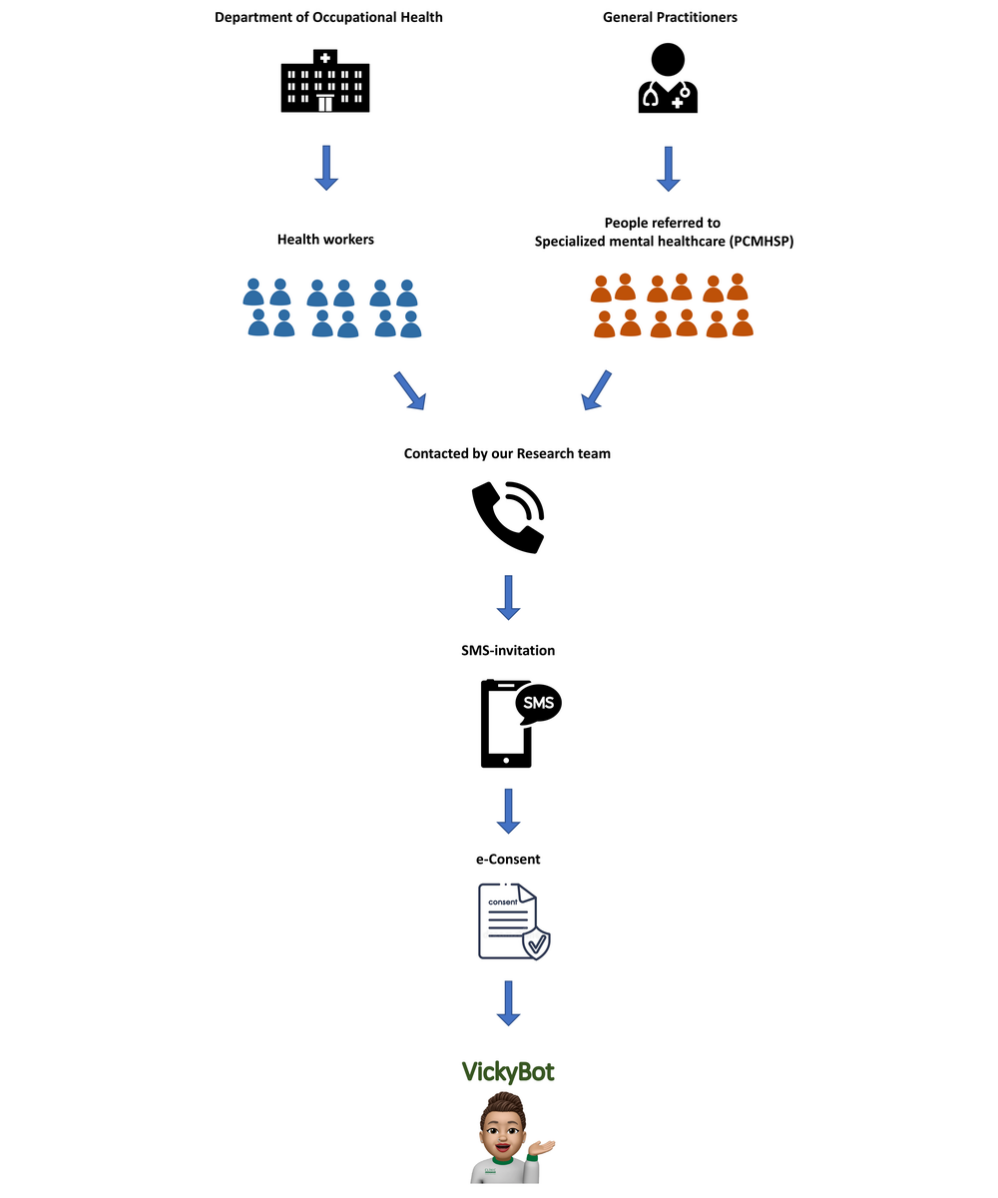
Recruitment procedure for the feasibility and potential effectiveness study.

Participation in the study was offered during a clinical consultation with either the GP (for people referred to the PCMHSP) or the Department of Occupational Health (for health care workers). Subsequently, our research team contacted potential participants, explained the details and conditions of the study, and resolved doubts. If participants consented to participate, they received an SMS invitation with access to a survey from Hospital Clínic de Barcelona, where they signed the informed consent form for participation, facilitated sociodemographic and clinical data, and were given access to Vickybot (Figure 2). All the participants used their personal phones, and no economic compensation was provided for their participation.

After accessing Vickybot (Figure 1), the chatbot presented its functions and asked users to perform a baseline self-assessment of anxiety-depressive symptoms and work-related burnout. According to the results of the first self-assessment, a treatment plan consisting of psychological modules was generated for each user. In the following month, users were prompted to interact with the chatbot and perform their treatment plans sequentially using personalized reminders. Users were also advised to complete the self-assessments every 2 weeks by receiving a preconfigured reminder.

At the end of the feasibility and effectiveness study (1 month after the first self-assessment), similar to the simulation phase, users were prompted to assess their experience with the different functions of the chatbot. Subjective UEI (acceptability, usability, and satisfaction) was assessed using the MAUQ (Multimedia Appendix 2), and objective UEI (completion, adherence, compliance, and engagement) was retrieved from the servers. The feasibility was assessed based on a combination of subjective and objective UEIs.

### Statistical Analyses

Statistical analyses were conducted using IBM SPSS Statistics for Windows Version 28.0 (IBM Corp). The analyses of all subjects were considered until their last interaction with the chatbot. Sociodemographic and clinical data of the sample, as well as details on the general use of the intervention and the specific functions used, were evaluated using descriptive analyses. The Shapiro-Wilk test and visual inspection of *Q*-*Q* plots were used to assess whether continuous variables were normally distributed.

The main outcomes for potential effectiveness were changes in anxiety-depressive symptoms and work-related burnout measured with self-assessment scales using paired 2-tailed *t* tests or the Wilcoxon signed-rank test. Subgroup analyses were conducted to assess the influence of baseline symptoms and the effect of engagement on intervention effectiveness. In addition, to assess the relationship between use and effectiveness, we performed Pearson correlations on the number of times that modules were completed and the days of use with the change in clinical scales’ scores. The results of the MAUQ at the end of the first month of use (acceptability, usability, and satisfaction), as well as other objective-use UEIs (completion, adherence, compliance, and engagement) and feasibility, were presented using descriptive analyses and analyzed according to the definitions recently proposed by the International Society for Bipolar Disorders Big Data Task Force [72]. Statistical significance was set at 2-tailed *P*≤.05.

### Ethics Approval and Consent for Publication

The PRESTO project was approved by the Clinical Research Ethics Committee at the Hospital Clínic of Barcelona (HCB/2020/0735). All data were collected and stored in encrypted and secure servers following the guidelines and standards of the 2018 European General Data Protection Regulation and were solely managed by project researchers. The project is in line with the ethical standards of European experts on personalized interventions and precision psychiatry [73]. All participants provided written informed consent before inclusion in the study.

## Results

### Setup Phase

A total of 40 HCs tested Vickybot simultaneously. They used different functions of the intervention, with all functions tested by at least 7 users. Data were transmitted and registered correctly, and the servers proved reliable with a rapidly increasing number of users.

### Simulation Phase

A total of 17 HCs (n=13, 76% female; mean age 36.5, SD 9.7 years) simulated different clinical situations while using Vickybot. Table 1 shows the specific clinical simulations and respective functions of the interventions to be assessed.

All simulators signed up, performed the first self-assessment, and obtained a treatment plan with specific psychological modules. According to the respective simulations, 98.8% of the expected modules were recommended to the users (Table 1). Only 1 module was not offered in the respective treatment plan (user 4) because it had a lower-than-expected score on the PHQ-9. Furthermore, 59% (10/17) of users were offered “extra” modules, which were not expected in the simulation. This was mostly because of high scores on the clinical scales by simulators who were asked to report specific symptoms solely (users 9-16). The expected simulated severity of anxiety and depressive symptoms was achieved by 75% of the users (1-4) with 1 scoring below the expected severity (user 4). In total, 98.5% of the expected functions (Table 1) were tested by simulators according to each clinical situation with the expected outcomes. All simulators showed a progressive reduction in scales’ scores according to the clinical simulation of symptom reduction. This was not the case for 1 user (user 17) who was instructed to simulate “erratic behavior.” Suicidal alerts were correctly activated by expected users (4 and 7) and received by the research team. Overall, 53% (9/17) of users set up “weekly objectives,” of which 67% (6/9) rated their daily completion. Moreover, 65% (11/17) of users set reminders, and 53% (9/17) of users registered an audio file after self-assessment.

A total of 65% (11/17) of HCs responded to the MAUQ after the simulation phase, with a mean score of 6.39 (SD 0.36) on a scale from 1 (strongly disagree) to 7 (strongly agree). All simulators (34/34, 100%) agreed with 14 of 18 statements of the MAUQ questionnaire. Most patients (72%-90%) agreed with the remaining 4 of the 18 statements. Vickybot showed high usability, satisfaction, and acceptability among the simulators. The most recurrently perceived weak points to be improved were the lack of reminders, the user’s lack of capacity to configure or personalize them, the lack of incentives to answer the self-assessments or access the psychological modules, and not being understood by the chatbot many times.

### Feasibility and Effectiveness Study

#### Overview

More than 300 patients (>150 from PC and >150 from health care workers) were offered participation in the study by their GPs or personnel from the Department of Occupational Health, respectively. After being contacted and receiving instructions from our research team, 130 patients (55 from PC and 75 health care workers) received an SMS invitation. A total of 34 patients (15 from PC and 19 from health care workers; 26.2% (34/130) of those who received the SMS invitation) signed up and performed the first self-assessments, with 100% (34/34) of patients presenting with low-to-severe anxiety symptoms, 94% (32/34) with depressive symptoms, and 65% (22/34) reported work-related burnout.

Table 2 provides a summary of the sample characteristics. Most of the patients were female (26/34, 77%). Mean age was 35.3 (SD 10.1) years. A minority of participants used alcohol or cannabis (<6%). More than 30% of the patients had medical comorbidities (17/34, 47%) or were prescribed nonpsychiatric drugs (13/34, 38%). Overall, 88% (30/34) had current psychiatric diagnoses, most commonly anxiety (18/34, 53%), followed by adjustment and depressive disorders (7/34, 21%), and 82% (28/34) had currently prescribed psychiatric drugs.

A total of 56% (19/34) of patients used Vickybot for several days, with a mean of 15.3 (SD 17.6) days of use. Furthermore, 27% (9/34) of patients completed the second self-assessment after 2 weeks of use, and 6% (2/34) completed the third self-assessment after 4 weeks of use. The longitudinal self-assessment scores are presented in Table 3. The Shapiro-Wilk test and visual examination of the *Q*-*Q* plots showed that the GAD-7 and PHQ-9 scores had a normal distribution, whereas the distribution of work-related burnout scores deviated from normality.

**Table 2:** Characteristics of the sample (N=34).

Sociodemographic variables				Value
Age (years), mean (SD)				35.3 (10.12)
Sex (female), n (%)				26 (77)
**Medical record (yes), n (%)**				
	Medical comorbidities			17 (47)
	Nonpsychiatric drugs prescribed			13 (38)
**Current drug use (yes), n (%)**				
	Cannabis			2 (6)
	Alcohol			1 (3)
**Mental health record, n (%)**				
	Past psychiatric diagnosis			11 (29)
	**Current psychiatric diagnosis^a^**			30 (88)
		Anxiety disorders	18 (53)	
		Adjustment disorders	7 (21)	
		Depressive disorders	7 (21)	
		Personality disorders	1 (3)	
		Eating disorders	1 (3)	
**Psychiatric drugs prescribed, n (%)**				
	Past psychiatric drugs prescribed			6 (18)
	**Current psychiatric drugs prescribed**			28 (82)
		Antidepressants	23 (68)	
		Benzodiazepines	10 (29)	
		Trazodone or mirtazapine	6 (18)	
		Low-dose amitryptiline	2 (6)	
		Other	4 (12)	

^a^Diagnoses were of low to moderate severity.

**Table 3:** Longitudinal self-assessment scores.

	First (baseline) (n=34)	Second (2 weeks) (n=9)	Third (4 weeks) (n=2)
GAD-7^a^, mean (SD)	13.4 (4.9)	13.0 (4.7)	10.5 (6.4)
PHQ-9^b^, mean (SD)	13.2 (6.6)	12.7 (7.4)	11.0 (7.1)
Work-related burnout, median (IQR)	2.0 (0.25-2.0)	0 (0.0-1.52)	0 (—^c^)

^a^GAD-7: Generalized Anxiety Disorder 7-item.

^b^PHQ-9: Patient Health Questionnaire-9.

^c^Not available.

#### Potential Effectiveness of the Intervention

No significant differences between the means of the first and second self-assessments were found for depressive (*t*_8_=0.40; *P*=.70) or anxiety (*t*_8_=1.00; *P*=.34) symptoms. This lack of significant difference was maintained for the third self-assessment. Work-related burnout scores were significantly lower after 2 weeks of using the Vickybot (*z*=−2.07; *P*=.04), with a moderate effect size (*r*=0.32; Table 3).

To assess the effect of baseline symptoms, we conducted subanalyses of participants with mild-to-moderate depression (PHQ-9, 5-14) and anxiety (GAD-7, 5-14) symptoms. No significant differences were found in depressive (*t*_4_=0.33; *P*=.75) or anxiety (*t*_4_=0.00; *P*=.99) symptoms between the means of the first and second self-assessments.

To assess whether the effect of the intervention was influenced by the use of the chatbot (dose-related effects of the intervention), we correlated the number of times that modules were performed (active treatment) with the change in the clinical scales’ scores. No significant associations were found for depressive symptoms (*r*=−0.48; *P*=.16), anxiety symptoms (*r*=0.12; *P*=.76), or work-related burnout (*r*=0.55; *P*=.13). We also correlated the “days using the chatbot” with the change in the clinical scales’ scores, and no significant associations were found for depressive symptoms (*r*=−0.23; *P*=.58), anxiety symptoms (*r*=−0.41; *P*=.36), or work-related burnout (*r*=0.38; *P*=.39). Despite the nonsignificant results, there was a trend toward a reduction in anxiety and depressive symptoms with greater chatbot use. To assess the effect of “clinically significant doses” of the intervention, we conducted subanalyses of patients with ≥50% (moderate to high) engagement. No significant differences between the scores of the first and second self-assessments were found for depressive symptoms (*t*_6_=−0.82; *P*=.44), anxiety symptoms (*t*_6_=0.33; *P*=.75), or work-related burnout (*z*=−1.89; *P*=.06).

#### Functions Used

All patients obtained a treatment plan with specific psychological modules that were recommended based on the first self-assessment. A total of 74% (25/34) of patients had access to their treatment plans and had performed at least one psychological module. In total, 112 modules were performed by users (mean 4.5, SD 2.4). The most frequently performed modules were depressed mood and anxiety (32 and 31 times, respectively), followed by work-related stress and burnout (17 and 15 times, respectively). Modules corresponding to depressive cognition; suicidal thoughts, decreased concentrations and irritability were not performed by any of the users. Within the modules, 100% (34/34) of patients who accessed the modules performed psychoeducation, 41% (14/34) the weekly objective, 24% (8/34) sense-based therapy, 18% (6/34) mindfulness, and 6% (2/34) metaphors. Moreover, 24% (8/34) of patients set up reminders for their “weekly objective,” and 50% (4/8) rated their daily completion; 65% (22/34) of patients set a reminder, 41% (14/34) registered an audio file after the first self-assessment, and 6% (2/34) registered new audio files after the second and third self-assessments.

Overall, 9% (3/34) of patients had activated suicide alerts. Vickybot recommended that they go to the psychiatric emergency department and provided emergency phone numbers, and the research team contacted them by phone call within 8 hours. All 3 patients responded to and acknowledged the call. Before the call, 1 of the contacted patients had overdosed on benzodiazepines and had suicidal intentions; therefore, he was urgently referred to the emergency department with a successful outcome. The other 2 contacted patients were referred to outpatient specialized mental health care services, as their current suicidal risk was deemed low by the clinical evaluation.

#### User-Engagement Indicators

With regard to subjective UEIs, 24% (8/34) patients responded to the MAUQ (Multimedia Appendix 2) 1 month after using Vickybot, with a mean score of 6.53 (SD 0.34) on a scale from 1 (strongly disagree) to 7 (strongly agree). All patients (34/34, 100%) agreed to 15 of 18 statements from the MAUQ questionnaire. Most patients (27/34, 80%) agreed with the remaining 3 of the 18 statements. All patients agreed on the 12th MAUQ statement, evaluating “satisfaction” with the chatbot, and also on the 18th statement, evaluating the “acceptability” of the intervention. Vickybot showed high usability, satisfaction, and acceptability. The most usefully perceived functions of the chatbot were (in order of rating from best to worst): “self-assessments,” “personalized treatment plan,” “detection of suicidal thoughts,” “contents of the psychological modules,” and the “weekly objective.”

Concerning objective UEIs, 24% (8/34) patients completed the study at 1 month, highlighting a low “completers rate.” Low adherence was also observed as only 6% (2/34) of patients used the chatbot after 4 weeks. Patients used the chatbot actively for 42.5% days of those expected (30 days), thus showing a low “compliance.” Patients used the chatbot actively for 23% (42/136) of the expected weeks (at least 5-7 days of each week, out of 4 weeks expected), meaning that “engagement” was also low. Overall, the results showed that Vickybot had low completion rates, adherence, compliance, and engagement.

Conversely, Vickybot showed high subjective UEIs (usability, satisfaction, and acceptability) but low objective-UEI (completion, adherence, compliance, and engagement). Considering the contrast between subjective and objective UEIs (perceived usefulness and actual use, respectively), Vickybot was deemed moderately feasible.

## Discussion

### Principal Findings

This study details the development and evaluation of the feasibility and potential effectiveness of a chatbot for anxiety, depressive symptoms, and work-related burnout among both patients from PC and health care workers. This intervention forms part of a larger project that aims to generate a digital platform (PRESTO) combining machine-learning severity prediction models with a smartphone-based app for screening, monitoring, and delivering psychological interventions [56]. The smartphone-based intervention has been envisaged to cover some of the most concerning demands from the PC system and also to provide personalized help to health care workers, as both have been affected by the COVID-19 pandemic. These multiple purposes require stepwise and solid growth as well as the collaboration and coordination of specialists in different areas.

The chatbot was useful in screening anxiety and depressive symptoms and work-related burnout in patients from PC and health care workers using self-administered scales. Although there were no statistically significant reductions in anxiety or depressive symptoms in the follow-up self-assessments, there was a significant reduction in work-related burnout (present in >60% of our sample). Similar to other digital solutions [74,75], Vickybot pointed toward potential effectiveness in reducing work-related burnout. Further research with increased statistical power is required to confirm these findings. There is a need to tackle this growing issue with measures from occupational and health care systems, and digital tools could offer a potential solution to this highly prevalent problem. Finally, the chatbot allowed for accurate detection and prompt intervention in emergency situations (suicidal thoughts).

It is known that health care workers and people referred to specialized mental health care from PC are distinct in several aspects, including medical literacy and work-related conditions. Despite the differences between both groups, they also share several common aspects, such as a high prevalence of depressive and anxious symptoms. Even though the causes of work-related burnout are particularly and highly specific (ie, stress and lack of support at the workplace), its manifestations include mostly anxiety and depressive symptoms such as fatigue, insomnia, and sadness. Moreover, even though health care workers share a common work environment, they are highly heterogeneous in their backgrounds and work situations, including people from medical staff, nursing staff, nursing assistants, administrative workers, management, cleaning staff, security, and logistical support. In fact, during the development phase, these considerations were debated and taken into account, but we found that despite the distinct characteristics between the 2 groups, their problems and preferences for a mental health app were highly similar. In this study, subanalyses between health care workers and PC patients regarding UEIs and potential effectiveness outcomes were performed, but no differences were found. This finding reinforces the hypothesis that these 2 groups have more common mental health problems than specific differences.

It should be noted that severe anxiety and depressive symptoms (GAD-7 or PHQ-9 ≥15) were observed in 44% (15/34) and 42% (14/34) of the patients, respectively, and 24% (8/34) presented both severe anxiety and depression simultaneously. In addition to the reduced sample size and low engagement, the high severity of symptoms in >40% of the sample may explain the lack of reduction in anxiety and depression symptoms. Digital interventions have only been found to be effective in the reduction of mild-to-moderate depressive symptoms [41], but not for severe symptoms. Therefore, consideration of the characteristics of the target population is of utmost importance when designing digital interventions. In our case, we managed to identify patients with severe anxiety and depression, which, it could be argued, are probably not suited for this kind of intervention, as they may require face-to-face visits with mental health specialists. In this sense, according to the literature, chatbots may be useful screening tools for mental health symptoms by providing a “humanized” environment while preserving anonymization [76,77].

Detecting patients with high severity is a core problem faced by overloaded PC systems. Nowadays, the prioritization system has been shown to be ineffective [78], and digital solutions that can screen for severity may be suitable for solving this problem. Vickybot was useful and accurate in detecting both patients with higher severity in addition to people with suicidal thoughts, while also offering on-time tools for immediate help (ie, emergency services) and alerting the research team to evaluate and propose tailored interventions. Suicide is probably the most striking unsolved mental health problem [79], and COVID-19 consequences are likely to worsen this situation [80-83]. Suicidal behaviors are the most alarming emergency situations that can be encountered; thus, tools that allow prompt detection and immediate interventions are crucial. Digital solutions may fulfill these requirements [84], with some interventions already showing positive evidence [85]. In fact, because this is a very sensitive issue, consensus statements on how to tackle suicide with digital technologies have recently come to light [86], and conclusive remarks regarding the future consensus on the applicability of digital solutions in mental health in both research and clinical practice are yet to come.

Some smartphone-based interventions have shown efficacy in outcomes such as well-being, quality of life, or perceived stress, which are considered more important than symptom reduction by patients [87]. Likewise, interventions such as psychoeducation empowered patients with strategies to cope with symptoms. As such, even when symptoms are not reduced, enhancing patients’ perceptions of control and self-management are key interventions for improving their quality of life [88-90]. Studies on digital health interventions should therefore focus on these parameters and not only on symptom reduction to gain further insight into their effectiveness and real-world application.

Moreover, smartphone-based solutions usually include functions such as self-assessment of symptoms, longitudinal monitoring, detection of emergency situations (eg, suicidal thoughts), and specific tools based on psychological therapies (eg, CBT or mindfulness). It is still not clear which of the former functions are more suitable for each patient profile in terms of their severity of anxiety and depression, specific symptom presentation, or sociodemographic characteristics [91]. The subanalyses and correlations performed in this study showed a nonsignificant trend toward a reduction in anxiety and depressive symptoms and work-related burnout with greater use of the chatbot. However, the small sample size in this longitudinal assessment may have limited the results. Future studies with increased statistical power are required to shed light on the remaining unanswered questions.

Regarding UEIs, subjective perceptions of use (acceptability, usability, and satisfaction) were highly positive, both for HCs during the simulation phase and for patients during the feasibility and effectiveness studies. Patients especially valued the ability to perform mental health self-assessments and personalization of treatments, as well as the chatbot’s ability to detect urgent situations and offer immediate help. This contrasts with the low objective-use metrics (completion, adherence, compliance, and engagement). This was probably because the chatbot had a rigid and sequential structure that users had to follow (based on the usual clinical evaluation and treatment procedures), thus lacking flexibility of use, which is key to user retention. This was shown by the initial use of the chatbot on day 1, with all users completing the first self-assessments and accessing at least some modules of the treatment plan, followed by a gradual reduction using the chatbot, leaving many modules incomplete. Furthermore, some modules were not accessed by any user. Other factors that may have lowered engagement outcomes are probably the lack of incentives offered by the intervention (eg, paucity of personalized reminders) or the limitations of the natural-language understanding of the chatbot, as stated in the comments of the simulation phase. A decrease in engagement in the use of digital tools over time has been reported in the literature [48,92,93]. For instance, studies with similar digital interventions reported equivalent results on engagement, with most participants only using the app once or twice [94], or only 20% of participants providing the required information in the app during the study period, with a gradual loss of entries after day 1 [95]. This suggests that engagement is a widespread problem with digital interventions and that it is key to develop strategies to mitigate it. Interventions designed for brief interactions have shown higher engagement outcomes [96]. This suggests the need to adapt smartphone-based interventions to the ways people use these technologies (usually briefly and concisely) [41]. On the basis of users’ comments and the evidence in the literature, we propose that future versions of the chatbot should aim to be more flexible by offering users alternative flows of use at all times and also include gamification with user incentives, adding personalization strategies to the psychological modules and reminders, and enhancing the chatbot’s interpretation and response capacities with NLP techniques.

### Limitations

First, the limited sample size of the feasibility and effectiveness study, the low engagement with the intervention, and the high severity of anxiety and depressive symptoms of many of the patients included may have precluded the possibility of finding the potential effectiveness of the intervention in reducing anxiety and depressive symptoms. Accordingly, further research with increased sample sizes, particularly of patients with mild-to-moderate anxiety and depressive symptoms, and longer follow-ups are required to determine the effectiveness of the intervention.

Second, we did not assess PTSD symptoms, which are prevalent among health care workers [23], and other relevant outcomes, such as well-being, quality of life, and perceived stress [87]. We suggest that future studies consider these aspects to achieve a holistic perception of the intervention by users.

Third, although Vickybot succeeded in cross-sectionally screening and establishing the severity of anxiety and depressive symptoms and work-related burnout, it failed to provide follow-up (biweekly) information on the evolution of most of the sample. Notably, obtaining cross-sectional information on symptom severity is key for successful triage in PC settings and for identifying emergency situations (eg, suicidal thoughts) in which the intervention was useful. However, the key goal of the PRESTO project is to provide self-management tools for people with anxiety and depressive symptoms. Moreover, only 26.2% (34/130) of patients who received the SMS invitation signed up and performed the first self-assessments. This represents a loss of almost 3 of 4 potential participants, which is in line with other studies showing similar losses of potential participants in the initial phases [94]. We believe that this was because of access and download difficulties, hence, the future versions of the chatbot should simplify this process as much as possible. To achieve this, in-app sign-up (eg, ensuring all steps can be conducted within the app itself) and reducing the amount of data required from participants before access may provide a solution to reduce the complexity of this process. Altogether, accessibility to the chatbot and its functions need to be redesigned considering user preferences while also keeping in mind the relevance of monitoring patient evolution.

Finally, there is still a lack of consensus on how to assess the feasibility of smartphone-based interventions, especially chatbots [88,97]. In addition, UEIs still require consistent and replicable standard definitions and valid measures with solid thresholds [98]. In this study, we applied the definitions and metrics recommended by the International Society for Bipolar Disorders [72]. Although these guidelines are based on digital interventions for bipolar disorder, most concepts may be applicable to other types of interventions. In recent years, platforms and databases of mental health smartphone-based interventions have been developed [99,100], including information on the characteristics of the intervention, functions included, population for whom they are directed, evidence-based validation, professional support, and expert reviews. Future directions point toward international expert consensus, providing homogeneous valid indications on digital solutions’ regulation [101] and clinical validation [102].

Future lines of the PRESTO project include (1) enhancing the chatbot NLP component, including natural-language understanding and generation capacities; (2) integrating the chatbot into a smartphone app to facilitate accessibility and incorporate the aforementioned functions; (3) performing focus groups with patients and professionals to detect needs and points of improvement to increase engagement; and finally (4) integrating the app into a digital platform to be implemented in the PC system.

### Conclusions

The chatbot was useful in screening for the presence and severity of anxiety and depressive symptoms. Although anxiety and depressive symptoms were not significantly reduced, there were significant reductions in work-related burnout on follow-up self-assessments, thus suggesting the potential effectiveness of Vickybot. Emergency situations were accurately identified and prompt interventions with successful outcomes were provided. Subjective perceptions of use (acceptability, usability, and satisfaction) were high, in contrast to low objective-use metrics (completion, adherence, compliance, and engagement), and the feasibility of the intervention was moderate. Our results are promising but suggest the need to adapt and enhance the smartphone-based solution to improve engagement. Finally, a consensus on how to report user engagement indicators and validate digital solutions, especially for chatbots, is required.

## Appendix

Multimedia Appendix 1 Scheme of the main functions of the intervention Vickybot.

Multimedia Appendix 2 Mobile Health App Usability Questionnaire for stand-alone mobile health apps used by patients.

## Abbreviations

CBT: cognitive behavioral therapy
GAD-7: Generalized Anxiety Disorder 7-item
GP: general practitioner
HC: healthy control
MAUQ: Mobile Health App Usability Questionnaire
NLP: Natural Language Processing
PC: primary care
PCMHSP: Primary Care Mental Health Support Program
PHQ-9: Patient Health Questionnaire-9
PRESTO: primary care digital support tool in mental health
PTSD: posttraumatic stress disorder
UEI: user-engagement indicator

## Data Availability

The data supporting the findings of this study are available upon request from the corresponding author.
