# Supplementary figures and images for: Vickybot, a Chatbot for Anxiety-Depressive Symptoms and Work-Related Burnout in Primary Care and Health Care Professionals: Development, Feasibility, and Potential Effectiveness Studies
Source: J Med Internet Res. 2023 Apr 3;25:e43293. doi: 10.2196/43293 (PMC10131622; doi:10.2196/43293)

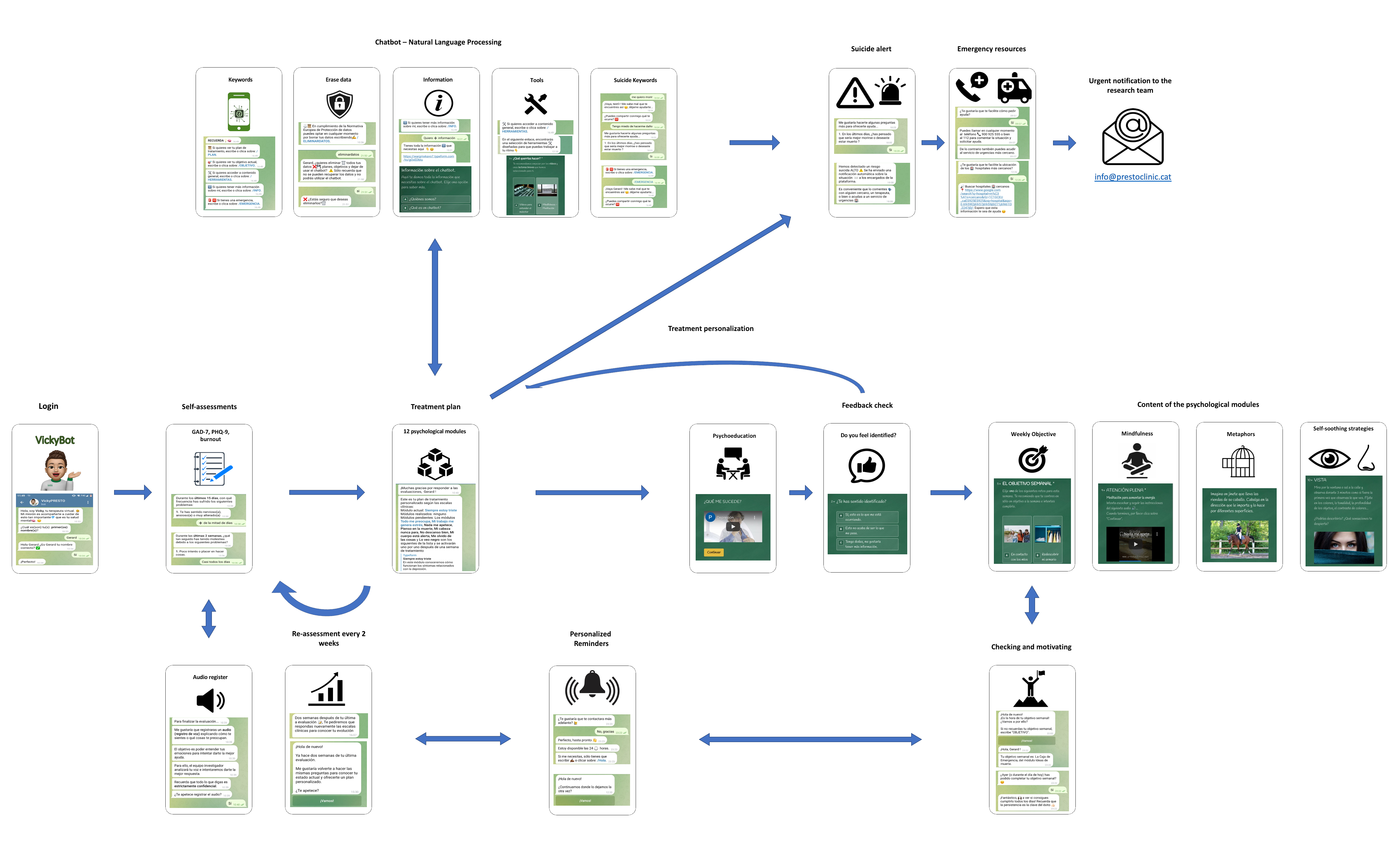

Supplement: Multimedia Appendix 1 [file jmir_v25i1e43293_app1.png]
