# Supplementary material for: Vickybot, a Chatbot for Anxiety-Depressive Symptoms and Work-Related Burnout in Primary Care and Health Care Professionals: Development, Feasibility, and Potential Effectiveness Studies
Source: J Med Internet Res. 2023 Apr 3;25:e43293. doi: 10.2196/43293 (PMC10131622; doi:10.2196/43293)
Supplement: Multimedia Appendix 2 [file jmir_v25i1e43293_app2.docx]

### Multimedia Appendix 1

## Table MA1 – mHealth App Usability Questionnaire (MAUQ) for Standalone mHealth Apps Used by Patients.

| Nº | Statement |
| --- | --- |
| 1. | The app was easy to use. |
| 2. | It was easy for me to learn to use the app. |
| 3. | The navigation was consistent when moving between screens. |
| 4. | The interface of the app allowed me to use all the functions (such as entering information, responding to reminders, viewing information) offered by the app. |
| 5. | Whenever I made a mistake using the app, I could recover easily and quickly. |
| 6. | I like the interface of the app. |
| 7. | The information in the app was well organized, so I could easily find the information I needed. |
| 8. | The app adequately acknowledged and provided information to let me know the progress of my action. |
| 9. | I feel comfortable using this app in social settings. |
| 10. | The amount of time involved in using this app has been fitting for me. |
| 11. | I would use this app again. |
| 12. | Overall, I am satisfied with this app. |
| 13. | The app would be useful for my health and well- being. |
| 14. | The app improved my access to healthcare services. |
| 15. | The app helped me manage my health effectively. |
| 16. | This app has all the functions and capabilities I expected it to have. |
| 17. | I could use the app even when the Internet connection was poor or not available. |
| 18. | This mHealth app provides an acceptable way to receive healthcare services, such as accessing educational materials, tracking my own activities, and performing self-assessment. |

The MAUQ includes 18 statements that evaluated from 1 to 7: 1 - strongly disagree, 2 – disagree, 3 – somewhat disagree, 4 – neither agree nor disagree, 5 – somewhat agree, 6 – agree, 7 – strongly agree. Afterwards, the average of response to all statements is a calculated to determine the usability of a mHeatlh intervention. The higher the overall average, the higher the usability. However, the MAUQ does not provide a cutoff point for considering an app “usable”, and only states that “the higher the overall average, the higher the usability of the app”. This scale was designed based on a number of existing questionnaires used in previous mobile app usability studies, including questionnaires focused on acceptability (Technology Acceptance Model (TAM) questionnaire [1]), usability (System usability scale (SUS) [2]), and satisfaction. In fact, the 12^th^ statement evaluates “satisfaction”, and the 18^th^ statement evaluates “acceptability” of the intervention. Four versions of the MAUQ are available considering the type of app (interactive or standalone) and target user of the app (patient or provider). This questionnaire has simplified the existing literature on user-engagement indicators (UEI), is adapted to mental health apps of different characteristics, and provides a web service for customization [3]. For the current study, we used the MAUQ for Standalone mHealth Apps Used by Patients.

1. Davis FD. Perceived usefulness, perceived ease of use, and user acceptance of information technology. MIS Q Manag Inf Syst Management Information Systems Research Center; 1989;13(3):319–339. doi: 10.2307/249008

2. Brooke J, others. SUS-A quick and dirty usability scale. Usability Eval Ind London--; 1996;189(194):4–7.

3. PITT Usability Questionnaire. Available from: https://ux.hari.pitt.edu/v2/portal/#/ [accessed Feb 1, 2022]
